# BLM prevents instability of structure-forming DNA sequences at common fragile sites

**DOI:** 10.1371/journal.pgen.1007816

**Published:** 2018-11-29

**Authors:** Hailong Wang, Shibo Li, Huimin Zhang, Ya Wang, Shuailin Hao, Xiaohua Wu

**Affiliations:** 1 Beijing Key Laboratory of DNA Damage Response and College of Life Science, Capital Normal University, Beijing, China; 2 Department of Molecular Medicine, The Scripps Research Institute, La Jolla, California, United States of America; University of Washington School of Medicine, UNITED STATES

## Abstract

Genome instability often arises at common fragile sites (CFSs) leading to cancer-associated chromosomal rearrangements. However, the underlying mechanisms of how CFS protection is achieved is not well understood. We demonstrate that BLM plays an important role in the maintenance of genome stability of structure-forming AT-rich sequences derived from CFSs (CFS-AT). BLM deficiency leads to increased DSB formation and hyper mitotic recombination at CFS-AT and induces instability of the plasmids containing CFS-AT. We further showed that BLM is required for suppression of CFS breakage upon oncogene expression. Both helicase activity and ATR-mediated phosphorylation of BLM are important for preventing genetic instability at CFS-AT sequences. Furthermore, the role of BLM in protecting CFS-AT is not epistatic to that of FANCM, a translocase that is involved in preserving CFS stability. Loss of BLM helicase activity leads to drastic decrease of cell viability in FANCM deficient cells. We propose that BLM and FANCM utilize different mechanisms to remove DNA secondary structures forming at CFS-AT on replication forks, thereby preventing DSB formation and maintaining CFS stability.

## Author summary

Common fragile sites (CFSs) are large chromosomal regions which are more prone to breakage than other places in the genome. They are a part of normal chromosome structure and are present in all human beings, but are also hotspots for chromosomal rearrangement during oncogenesis. Understanding how CFSs are protected to prevent genome instability is thus extremely important for revealing the mechanism underlying cancer development. We found that Bloom syndrome protein BLM is involved in resolving DNA secondary structures that arise at AT-rich sequences in CFSs, suggesting a critical function of BLM in protecting CFSs. We also found that this BLM function is distinct from the role of Fanconi anemia protein FANCM in protecting CFSs, and loss of both BLM and FANCM activities leads to cell death. These studies reveal important mechanisms of the maintenance of CFS stability in mammalian cells.

## Introduction

Genome instability is a hallmark of cancer cells [1]. Certain chromosomal loci, such as CFSs are hotspots for genome instability and are prone to chromosomal rearrangement [2]. CFSs are part of normal chromosomal regions that are present in all individuals, but are more susceptible to breakage than other genome loci under replication stress [3]. CFSs are preferential sites for sister chromatid exchanges and viral DNA integrations [2, 4, 5]. They are associated with chromosomal breakpoints observed in cancer cells which often involve deletions of tumor suppressor genes and amplifications of oncogenes [6–11].

Multiple mechanisms have been proposed to elucidate replication stress-induced CFS breakage (often termed as CFS expression) [12]. CFSs tend to be replicated late, and often lack sufficient replication origins [13, 14]. Due to origin paucity at CFSs, all available origins are utilized under normal conditions and no more dormant origins can be activated upon replication stress, which leads to unfinished DNA replication in mitosis and chromosome breakage [14]. Meanwhile, CFSs often contain very large gene, which could induce collisions of replication and transcription machineries, contributing to CFS expression [15]. In addition to these mechanisms, CFSs contain AT-rich sequences which are predicted to form strong DNA secondary structures [16]. These AT-rich sequences can block DNA replication *in vitro* [16, 17]. Importantly, it has been shown that replication stalling indeed occurs *in vivo* at the AT-rich sequences derived from FRA16D when they are present in yeast, and also at the AT-rich sites in FRA16C and FRA16B in mammalian cells [18–20]. We observed that CFS-derived AT-rich sequences induce DNA double strand break (DSB) formation and homologous recombination (HR)-mediated mitotic recombination [21]. Therefore, these CFS-derived AT-rich sequences (CFS-AT) are genetically unstable, and thus are one of the major contributors to CFS instability. However, not much is known about how the genome stability at CFS-AT is maintained to prevent CFS breakage.

FANCM is an ATP-dependent branch-point translocase, which is mutated in Fanconi anemia (FA) patients [22]. We previously found that FANCM has a unique activity independent of the FA core complex and FANCD2/FANCI to preserve stability of AT-rich sequences derived from CFSs [23]. Upon replication stress, CFS-derived AT-rich sequences form secondary structures when single-stranded DNA (ssDNA) is exposed at replication forks, and FANCM uses its translocase activity to promote fork reversal to remove such secondary structures, thereby preventing DSB formation. Homologous recombination (HR) is one major pathway to repair DSBs formed upon replication fork collapse [24]. In accordance with this, proteins that are involved in HR, such as BRCA1 and CtIP are important for maintaining stability of AT-rich sequences and suppressing CFS expression [21].

Bloom’s syndrome (BS) is a genetic disorder that is associated with a wide range of abnormalities including growth retardation, immunodeficiency, genome instability and predisposition to cancer [25, 26]. The BS associated gene product, BLM helicase, is a member of RecQ family, which acts as a 3’ to 5’ DNA helicase [27, 28]. BLM unwinds a variety of DNA substrates including Holliday junction, forked duplex, D-loop and G-quadruplex (G4) DNA [27, 29, 30] and is involved in multiple pathways contributing to the maintenance of genome stability. BLM is associated with topoisomerase IIIα, RMI1 and RMI2 to form a BTR complex that removes the Holliday junction by a mechanism called dissolution, thereby preventing crossover and sister chromatin exchange [31, 32]. BLM functions together with DNA2 in a non-overlapping pathway of Exo1 to mediate long-range end resection to generate 3’ single-stranded DNA tail from a DSB [33, 34]. BLM is also required for ensuring complete sister chromatid decatenation and thus is important for the suppression of anaphase bridges and faithful chromosome segregation [35].

In this study, we identified a new role of BLM in preventing DSB formation at CFS-AT sequences in a manner dependent on BLM helicase activity and ATR-mediated phosphorylation of BLM. This BLM function is non-overlapping with the role of Fanconi anemia (FA) protein FANCM in the maintenance of CFS stability identified in our previous study [23]. We propose that BLM resolves DNA secondary structure of CFS-AT sequences using a mechanism that is different from that of FANCM, and functions together with FANCM to preserve the integrity of CFSs.

## Results

### BLM is important for suppressing mitotic recombination induced by AT-rich sequences derived from CFSs

**Fig 1:**
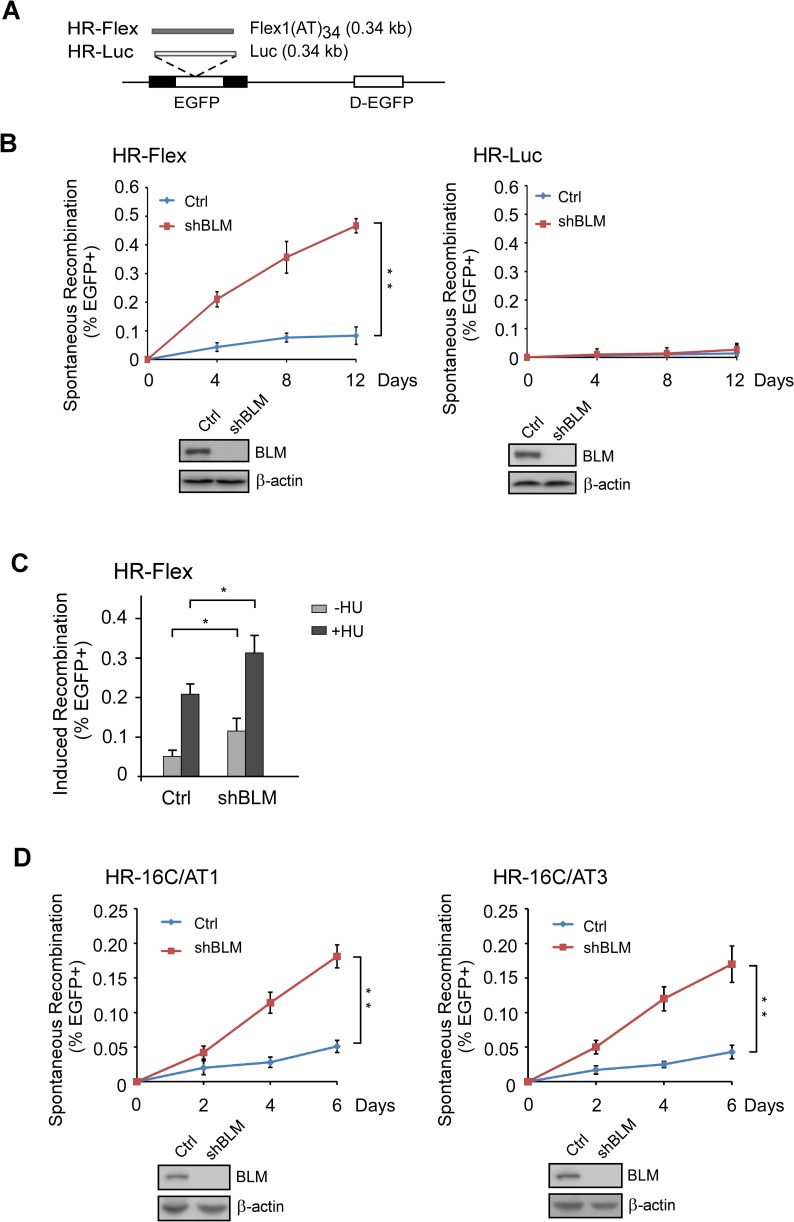
BLM is important for suppressing Flex1-induced mitotic recombination. In all experiments, error bars represent standard deviation (SD) of at least three independent experiments. The P value is indicated as *P<0.05 and **P<0.01, and n.s. is not significant. A. Schematic drawing of the EGFP-based HR-Flex and HR-Luc reporters as previously described [21]. Luc: luciferase fragment; D-EGFP: donor EGFP. B. U2OS cells containing the HR-Flex reporter (left) or HR-Luc reporter (right) were infected by lentiviruses encoding BLM shRNAs or a control vector (Ctrl), and spontaneous mitotic recombination was assayed on indicated days. The expression of BLM was examined by Western blot with β-actin as loading control. C. U2OS (HR-Flex) cells expressing BLM shRNA or vector (Ctrl) were generated by lentiviral infection followed by drug selection and two days later, cells were treated with 2 mM HU for 24 hr and assayed for EGFP-positive events 72 hr later. D. Spontaneous recombination was examined in U2OS (HR-16C/AT1) and U2OS (HR-16C/AT3) cells after culturing pre-sorted non-green cells for indicated days. 16C/AT1 and 16C/AT3 are AT rich sequences derived from FRA16C. The expression of BLM is shown by Western blot with β-actin as loading control.

As described previously, insertion of Flex1, the AT-rich sequence derived from FRA16D, into the EGFP-based HR reporter (HR-Flex) significantly increases mitotic recombination when comparing with the HR-Luc reporter which contains a luciferase sequence insertion of similar size [21] (Fig 1A). This HR-mediated mitotic recombination is caused by DSB formation at Flex1 due to Flex1 instability on replication forks, and in support of this, replication stress induced by hydroxyurea (HU) and aphidicolin (APH) enhances mitotic recombination at Flex1 [21]. Interestingly, suppression of BLM expression by shRNAs leads to increased mitotic recombination in U2OS (HR-Flex) cells (Fig 1B) [21]. Loss of BLM also increases HU-induced mitotic recombination (Fig 1C). These studies suggest that BLM plays a protection role in preventing instability of CFS-derived AT-rich sequences upon replication stress.

In addition to Flex1, AT-rich sequences derived from other CFSs also induce mitotic recombination [23]. When BLM is depleted by shRNAs, mitotic recombination induced by 16C/AT1 and 16C/AT3, two AT-rich sequences derived from FRA16C, is further increased (Fig 1D). This suggests that BLM-mediated protection is not limited to a specific AT-rich sequence but is a general mechanism to avoid instability at AT-rich sequences derived from CFSs.

### BLM is recruited to Flex1 and prevents DSB formation at Flex1

**Fig 2:**
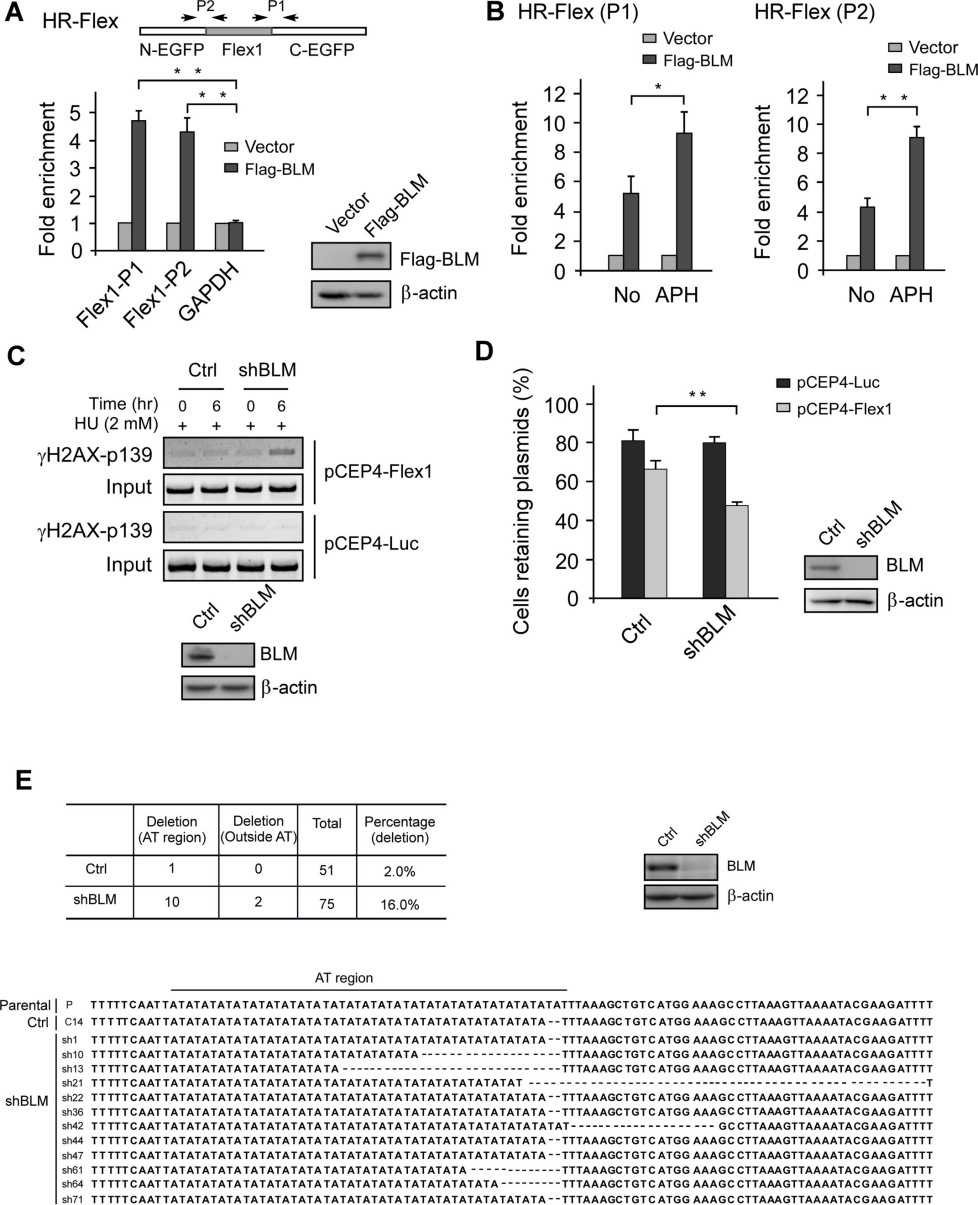
BLM is recruited to Flex1 to suppress DSB formation. A. Anti-Flag ChIP was performed in U2OS (HR-Flex) cells which contain stable chromosomal integrated Flex1 present in the HR-Flex reporter and express Flag-BLM or empty vector using primer sets (P1 or P2) as indicated for Flex1 with GAPDH as a control. Recovered DNA from anti-Flag ChIP was analyzed by qPCR. The expression of Flag-BLM is shown by Western blots. B. U2OS (HR-Flex) cells containing stable chromosomal integrated Flex1 and expressing Flag-BLM or empty vector were treated with or without APH (0.4 uM, 24 hr), followed by anti-Flag ChIP analyzed by qPCR using primer sets (P1 or P2) indicated in A. C. U2OS cells containing pCEP4-Flex1 or pCEP4-Luc plasmids were infected with lentiviruses expressing BLM shRNA or a vector control (Ctrl). Two days after shRNA lentiviral infection, cells were treated with HU (2 mM, 6 hr) and DSB formation at Flex1 or Luc surrounding regions was analyzed by ChIP analysis using H2AX-S139p (γH2AX) antibody, followed by regular PCR reaction. PCR products were resolved by 1.5% agarose DNA gel (top). The expression of BLM was examined by Western blot with β-actin as loading control (bottom). D. Plasmid stability assay was performed in U2OS cells carrying pCEP4-Flex1 or pCEP4-Luc plasmids after expressing BLM shRNAs or control vector (Ctrl). The expression of BLM was examined by Western blot analysis with β-actin as loading control. E. U2OS cells containing pCEP4-Flex1 plasmids were infected with lentiviruses expressing BLM shRNA or a control vector (Ctrl), and after culturing 10 days, plasmids were recovered and sequenced. The percentage of events containing deletions in Flex1 on pCEP4-Flex1 plasmids was calculated (top). Sequence alignment of the original Flex1 (P) and the Flex1 from recovered pCEP4-Flex1 plasmids that contain deletions are shown (bottom). Deletion (AT region): deletions are in the region of AT dinucleotide repeats in Flex1; Deletion (Outside AT): deletions extend to the regions outside of AT dinucleotide repeats.

To examine whether BLM is recruited to CFS-derived AT rich sequences, we performed chromatin immunoprecipitation (ChIP) of Flag-BLM to the Flex1 surrounding regions in the HR-Flex reporter which is stably integrated in the genome using two sets of primers P1 and P2 (Fig 2A). Flag-BLM is efficiently recruited to Flex1 in comparison to the GAPDH locus. APH treatment leads to further enrichment of Flag-BLM to Flex1 (Fig 2B). These data suggest that BLM is localized to Flex1 to suppress Flex1-induced mitotic recombination.

To examine whether BLM prevents DSB formation at CFS-derived AT-rich sequences upon replication stress, we performed ChIP analysis of γH2AX at Flex1 present on pCEP4-Flex1 plasmids with or without BLM depletion. γH2AX is significantly increased at Flex1 after exposure to HU when BLM is inactivated by shRNAs (Fig 2C and S1A Fig). We also performed ChIP analysis of γH2AX at endogenous FRA3B locus close to an AT-rich sequence and found that γH2AX is also significantly enriched there when BLM is depleted by shRNAs after APH treatment (S2 Fig). These data support the model that loss of BLM function induces DSB formation at AT-rich sequences in CFSs, thereby causing increased mitotic recombination at these sequences.

### Loss of BLM activity increases plasmid instability induced by Flex1

When we placed Flex1 in the Epstein-Barr virus (EBV) replication origin-containing plasmids (pCEP4-Flex1) that are propagated as episomes in mammalian cells, plasmid instability is increased comparing to the plasmids containing the luciferase control sequence (pCEP4-Luc) [21]. Plasmid instability of pCEP4-Flex1 is further increased when we knocked-down BLM by shRNAs (Fig 2D). We recovered pCEP4-Flex1 plasmids after propagating them in U2OS cells with or without expressing BLM-shRNAs for 10 days and sequenced Flex1. We found that deletions present at Flex1 on the pCEP4-Flex1 plasmids recovered from BLM-shRNAs expressing cells are significantly increased compared to that from control cells. While most deletions are present in the AT-dinucleotide region at Flex1, some deletions are extended outside of the AT-dinucleotide region (Fig 2E). These data support the idea that BLM plays an important role in protection of CFS-derived AT-rich sequences by preventing DSB formation. Instability of Flex1-containing plasmids observed in BLM depleted cells is likely caused by DSB formation and deletions present on the recovered pCEP4-Flex1 plasmids are likely the products of end joining at DSBs generated in Flex1.

### The helicase activity of BLM is important for the suppression of Flex1-induced mitotic recombination

**Fig 3:**
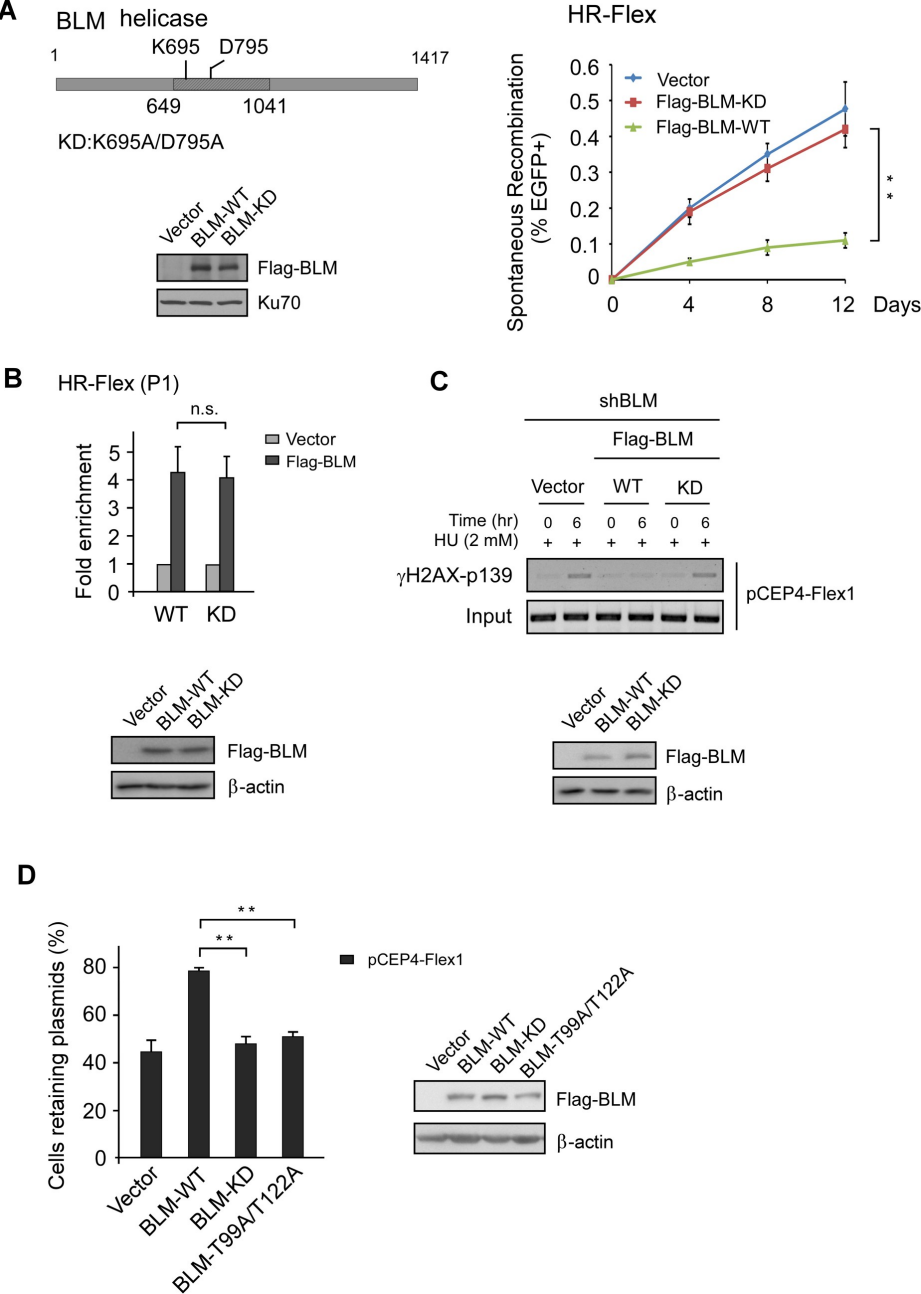
BLM helicase activity is important for preventing DSB formation and mitotic recombination at Flex1. A. Spontaneous recombination was assayed at indicated days in U2OS (HR-Flex) cells expressing Flag-BLM-WT or helicase mutant Flag-BLM-KD (K695A/D795A) after endogenous BLM was silenced by shRNA. The expression of Flag-BLM-WT and Flag-BLM-KD is shown by Western blot. B. Anti-Flag ChIP at Flex1 was performed in U2OS (HR-Flex) cells carrying chromosomal integrated Flex1 and expressing Flag-BLM-WT or helicase mutant Flag-BLM-KD with the vector as a control and analyzed by qPCR. The expression of Flag-BLM-WT and Flag-BLM-KD is shown by Western blot. C. Flag-BLM-WT or helicase mutant Flag-BLM-KD was expressed in U2OS cells carrying pCEP4-Flex1 plasmids with endogenous BLM silenced by shRNAs. Two days after shRNA expression, cells were treated with HU (2 mM, 6 hr) and DSB formation at Flex1 was analyzed by γH2AX ChIP of pCEP4-Flex1 plasmids with PCR products resolved on agarose gel (top). The expression of Flag-BLM-WT and Flag-BLM-KD is shown by Western blot (bottom). D. Plasmid stability assay was performed in U2OS cells reconstituted with Flag-BLM (WT, KD or T99A/T122A mutant) with endogenous BLM silenced by shRNA. The expression of Flag-BLM WT or indicated mutants is shown by Western blots.

BLM unwinds DNA secondary structures, such as G4 DNA [29]. We hypothesize that BLM uses its helicase activity to remove DNA secondary structures formed at AT-rich sequences, thus inhibiting DSB formation and mitotic recombination (See Discussion). We expressed Flag-tagged BLM wild-type allele (WT) and helicase mutant allele K695A/D795A (KD) [36] carrying silent mutations at the shRNA targeting sites in U2OS (HR-Flex) cells. Mitotic recombination was determined after delivery of shRNAs to inhibit the expression of endogenous BLM. BLM-WT, but not BLM-KD mutant suppresses Flex1-induced mitotic recombination (Fig 3A). Loss of BLM helicase activity does not influence its recruitment to Flex1 as revealed by ChIP analysis (Fig 3B). These data suggest that the BLM helicase activity is not required for its recruitment to Flex1, but is important for the suppression of Flex1-induced mitotic recombination.

To examine whether DSB formation is increased at Flex1 when BLM helicase activity is inhibited, we performed ChIP of γH2AX at Flex1 in U2OS (pCEP4-Flex1) cells expressing BLM-WT or BLM-KD helicase mutant. Indeed, loss of BLM helicase activity increases DSB formation at Flex1 (Fig 3C and S1B Fig). Loss of BLM helicase activity also increases pCEP4-Flex1 plasmid instability (Fig 3D). We propose that the BLM helicase activity is needed for unwinding DNA secondary structures formed at Flex1, which is important for preventing DSB formation at Flex1 and inhibiting Flex1-induced mitotic recombination (See Discussion).

### ATR-mediated phosphorylation of BLM is important for maintaining Flex1 stability

**Fig 4:**
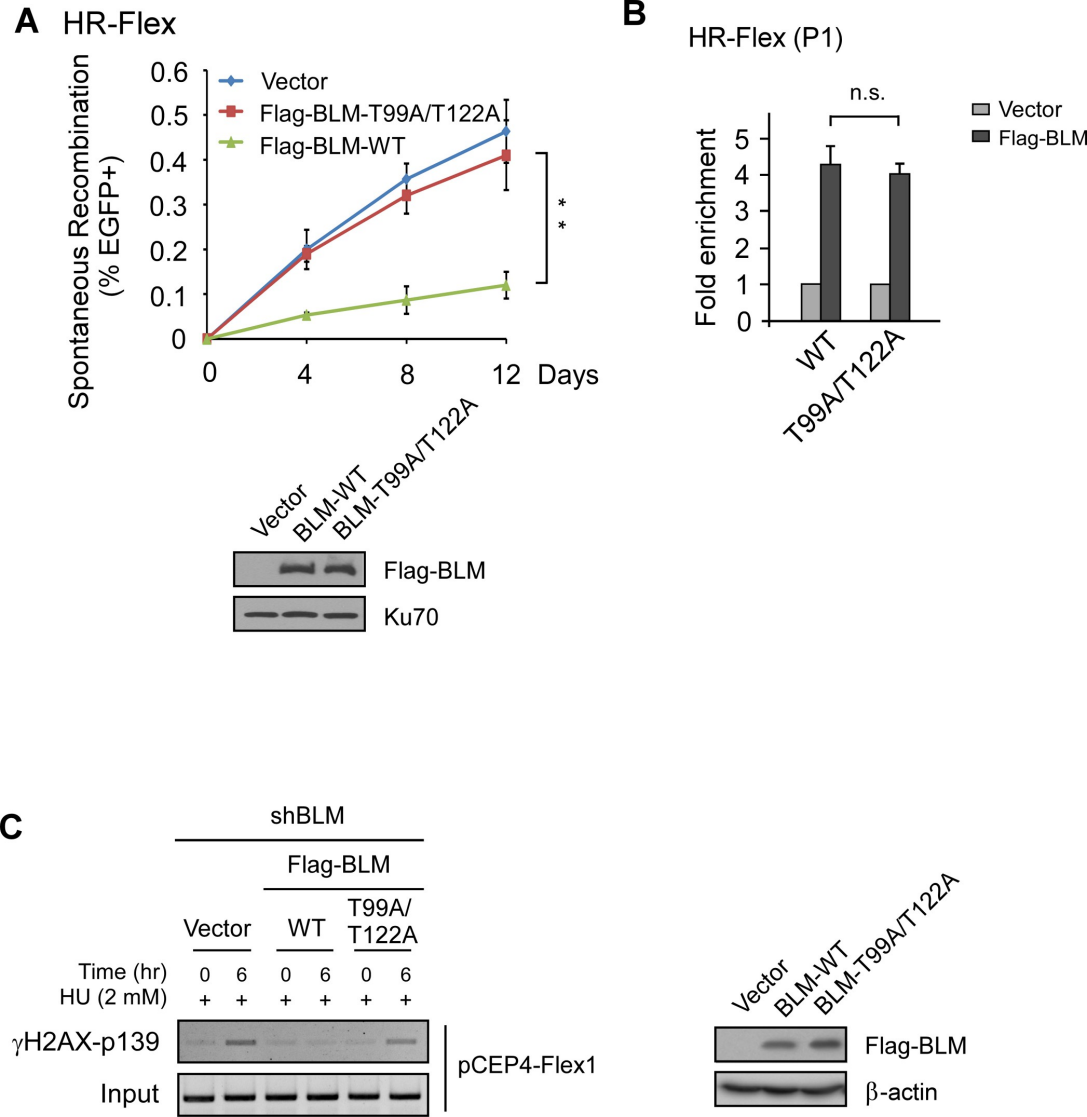
BLM phosphorylation by ATR was important for preventing DSB formation and mitotic recombination at Flex1. A. Spontaneous recombination was assayed at indicated days in U2OS (HR-Flex) cells expressing Flag-BLM-WT or Flag-BLM-T99A/T122A mutant after endogenous BLM was silenced by shRNAs. The expression of Flag-BLM WT and Flag-BLM-T99A/T122A is shown by Western blot. B. Anti-Flag ChIP at Flex1 was performed in U2OS (HR-Flex) cells carrying chromosomal integrated Flex1 and expressing Flag-BLM-WT or Flag-BLM-T99A/T122A mutant with the vector as a control. The expression of Flag-BLM-WT and Flag-BLM-KD is shown in A. C. Flag-BLM-WT or Flag-BLM-T99A/T122A mutant was expressed in U2OS cells carrying pCEP4-Flex1 plasmids with endogenous BLM silenced by shRNAs. Two days after shRNA expression, cells were treated with HU (2 mM, 6 hr) and γH2AX ChIP of Flex1 on pCEP4-Flex1 plasmids was performed with PCR products resolved on agarose gel (left). The expression of Flag-BLM-WT and Flag-BLM-KD is shown by Western blot (right).

BLM is phosphorylated by ATR at Thr99 and Thr122 sites upon replication stress [37]. To show whether ATR-mediated phosphorylation of BLM is important for suppressing Flex1-induced mitotic recombination, we expressed BLM-WT and BLM-T99A/T122A mutant in U2OS (HR-Flex) cells with endogenous BLM depleted by shRNAs. Spontaneous recombination at Flex1 is significantly higher in the BLM-T99A/T122A mutant cell line compared to that in the BLM-WT cell line (Fig 4A), suggesting that ATR-mediated phosphorylation of BLM is important for maintaining Flex1 stability. ChIP analysis showed that the recruitment of BLM-T99A/T122A mutant to Flex1 site is as efficient as that of BLM-WT (Fig 4B), indicating that ATR-mediated phosphorylation of BLM does not influence BLM recruitment to Flex1. However, ChIP of γH2AX showed that DSB formation is increased at Flex1 in the BLM-T99A/T122A mutant cell line compared to the BLM-WT cell line (Fig 4C and S1C Fig). pCEP4-Flex1 plasmid instability is also increased in the BLM-T99A/T122A mutant cell line (Fig 3D). These data suggest that ATR-mediated phosphorylation of BLM is important for protecting AT-rich sequences at CFSs to avoid DSB formation.

### BLM prevents Flex1 instability upon oncogene expression

**Fig 5:**
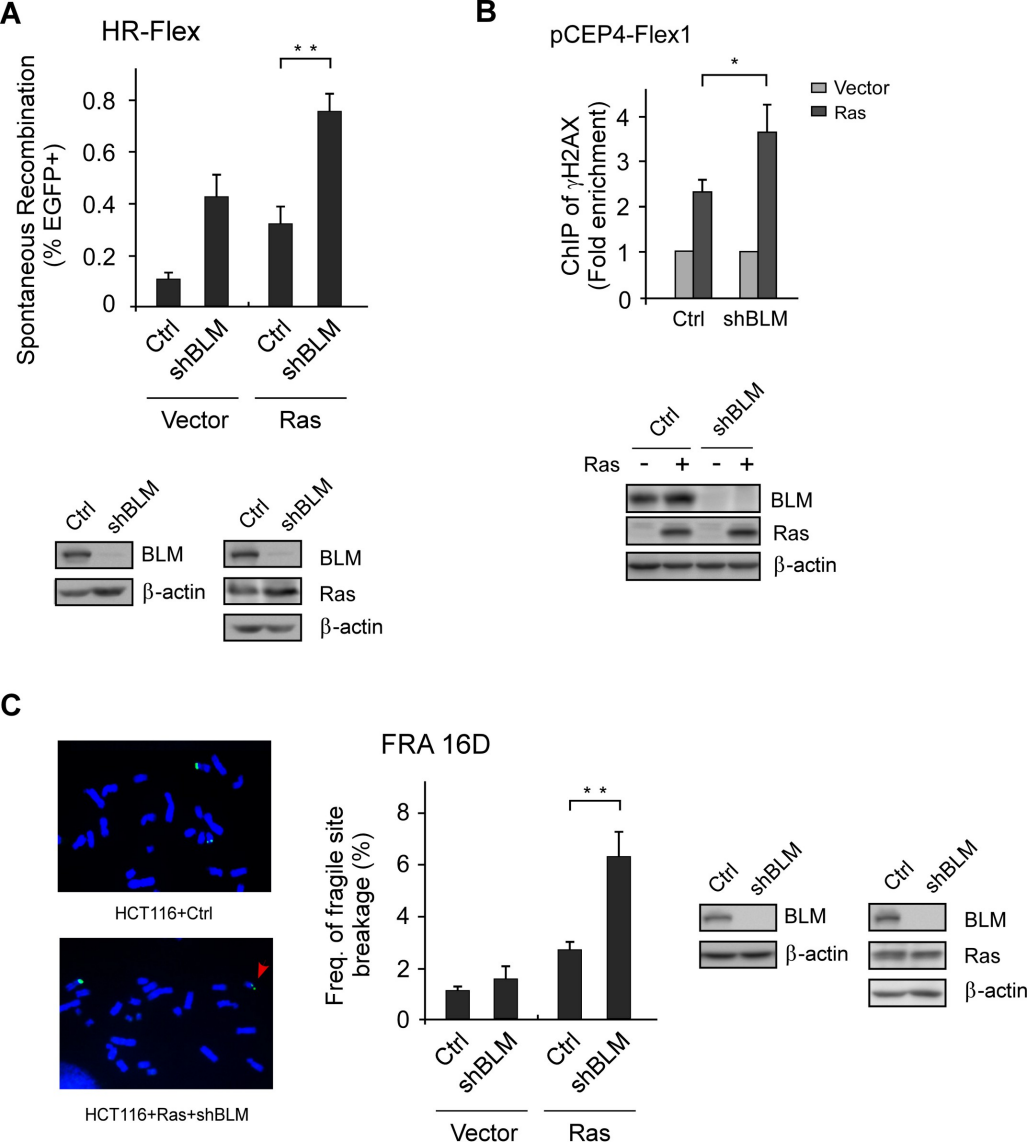
BLM is important for maintaining Flex1 stability and protecting CFSs upon oncogene expression. A. Spontaneous recombination was determined in U2OS (HR-Flex) cells expressing BLM shRNAs or control vector one week after expression of H-Ras V12 (Ras) or vector. The expression of BLM and Ras was examined by Western blot analysis with β-actin as loading control. B. U2OS (pCEP4-Flex1) cells were infected with lentiviruses expressing BLM shRNA or a control vector (Ctrl), followed by infection of Ras retroviruses or vector control. γH2AX ChIP at Flex1 on pCEP4-Flex1 plasmids was performed 3 days after Ras infection and analyzed by qPCR. ChIP value in vector Ctrl is set as 1 for normalization. C. Frequency of CFS expression at FRA16D in HCT116 cells expressing BLM shRNAs or control vector was determined after expressing Ras or vector. FISH images of metaphase chromosome spreads using probes to RFA16D are shown. The red arrow indicates the broken chromosome at FRA16D. The expression of BLM and Ras was examined by Western blot analysis with β-actin as loading control.

Flex1-induced mitotic recombination is increased upon HU treatment [21], which is further increased upon loss of BLM activity (Fig 1C). This suggests an important role of BLM in suppressing Flex1 instability upon replication stress. Since oncogene expression often leads to replication stress [38, 39], we examined whether BLM is important for maintaining Flex1 stability upon oncogene expression. Indeed, inactivation of BLM significantly increases mitotic recombination upon overexpression of H-Ras-V12 (Ras) (Fig 5A).

It has been shown that oncogene expression induces CFS expression [40]. We showed that γH2AX is significantly increased at Flex1 when Ras is expressed with a further increase when BLM is depleted by shRNAs (Fig 5B). By performing FISH analysis on mitotic chromosome, we showed that inactivation of BLM results in increased FRA16D expression after Ras expression (Fig 5C). Collectively, our study suggests that BLM is important for protecting AT-rich sequences at CFSs and prevents CFS expression upon replication stress induced by oncogene expression.

### The mechanism used by BLM to protect Flex1 is distinct from that used by FANCM

**Fig 6:**
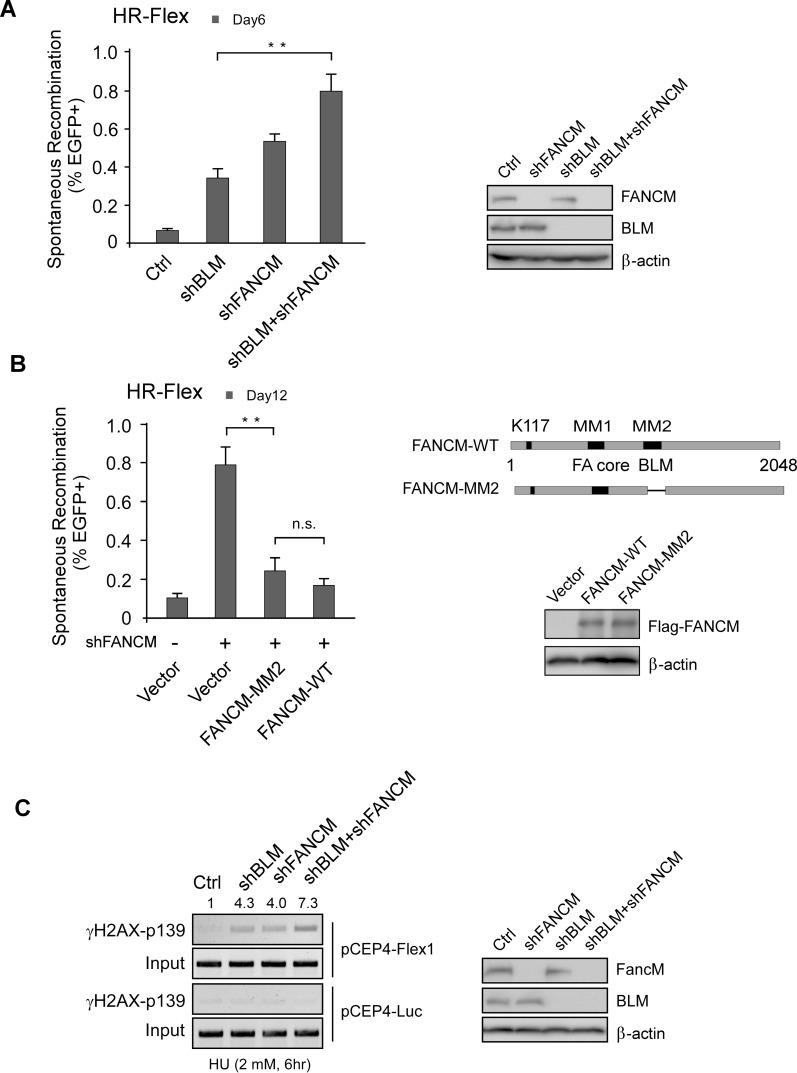
BLM and FANCM play distinct roles in protecting Flex1 stability. A. Spontaneous recombination was assayed in U2OS (HR-Flex) cells 6 days after infecting with lentiviruses encoding vector, BLM shRNA or FANCM shRNA alone or both. The expression of BLM and FANCM was examined by Western blot with β-actin as loading control. B. Spontaneous recombination was assayed in U2OS (HR-Flex) cells expression FANCM WT or MM2 mutant with endogenous FANCM silenced by shRNA. The expression of FLAG-FANCM WT or MM2 mutant was examined by Western blot analysis with β-actin as loading control. C. U2OS cells containing pCEP4-Flex1 or pCEP4-Luc plasmids were infected with lentiviruses expressing BLM shRNA or FANCM shRNA or both. Two days after shRNA lentiviral infection, cells were treated with HU (2 mM, 6 hr) and ChIP analysis of γH2AX at Flex1 or Luc on pCEP4-Flex1 or pCEP4-Luc plasmids was performed with PCR products resolved on agarose gel (left). The expression of BLM and FANCM was examined by Western blot with β-actin as loading control (right).

We showed previously that FANCM is important for maintaining Flex1 stability and its translocase activity is required for such activity [23]. Since BLM binds to FANCM through RMI1 [41, 42], we asked whether BLM protects Flex1 by modulating FANCM activity. We examined mitotic recombination at Flex1 when BLM and FANCM are inactivated individually or simultaneously (Fig 6A). Inactivation of BLM alone significantly increases Flex1-induced mitotic recombination, but the extent is less than that after FANCM inactivation. Simultaneous inactivation of BLM and FANCM causes hyper mitotic recombination at the level higher than inactivation of either BLM or FANCM alone. These data suggest that BLM and FANCM are not epistatic to each other for protecting Flex1 stability and that the mechanism utilized by BLM to protect Flex1 is not through the protection pathway mediated by FANCM. In consistent with a non-overlapping function of BLM and FANCM in protecting Flex1, the FANCM-MM2 mutant defective in BLM binding [S3 Fig, [41]] does not show a defect in suppression of Flex1-induced mitotic recombination compared to FANCM WT (Fig 6B). This suggests that the BLM function in protecting Flex1 is not through interacting with FANCM.

We also monitored DSB formation at Flex1 when BLM or FANCM is inactivated. ChIP analysis of γH2AX at Flex1 showed that inactivation of both BLM and FANCM causes more DSB formation than when either of them is inactivated after HU treatment (Fig 6C and S1D Fig). This study suggests that BLM plays a distinct role from FANCM and functions together with FANCM to prevent DSB formation at Flex1.

### Inactivation of BLM in FANCM deficient cells reduces cell viability

**Fig 7:**
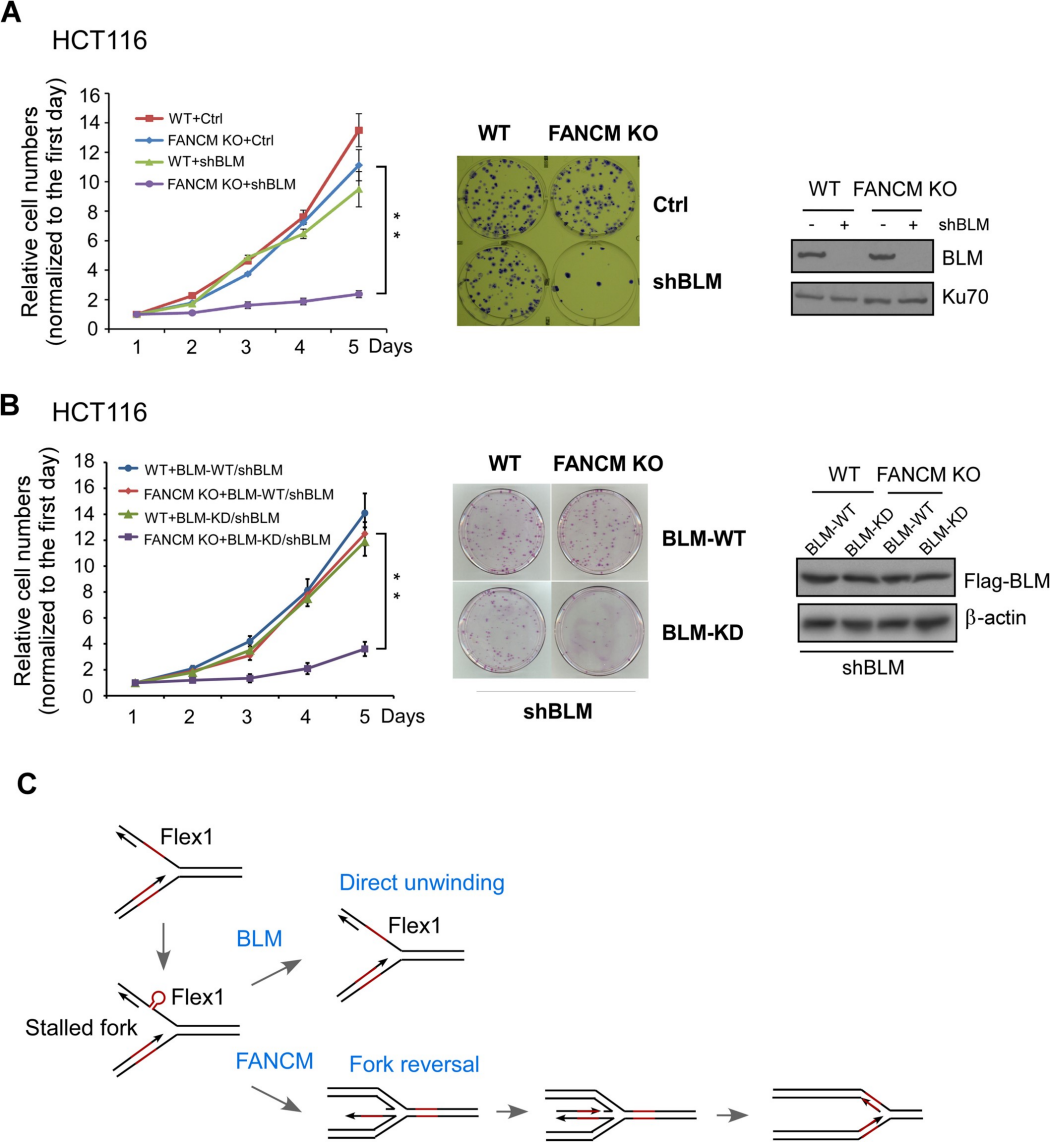
Synthetic lethality interaction of BLM and FANCM is also observed in non-ALT HCT116 cell line. A. Growth curve of HCT116 WT or FANCM KO cells was plotted after expressing BLM shRNA or control vector (left). The viability of cells was shown by colony formation assay (middle). The expression of BLM was examined by Western blot with Ku70 as loading control (right). B. Growth curve of HCT116 WT and FANCM KO cells reconstituted with BLM-WT or helicase KD mutant was plotted after endogenous BLM silenced by shRNA. The expression of FLAG-BLM WT or KD mutant was examined by Western blot analysis with β-actin as loading control. C. Proposed model for distinct roles of BLM and FANCM in protecting Flex1 (see text for details).

It was described that FANCM and BLM are recruited to telomeres in cells which specifically use the alternative lengthening of telomeres (ALT) mechanism to maintain telomere integrity, and depletion of BLM and FANCM leads to synthetic lethality in ALT cells [43]. These studies suggest a specific role of BLM and FANCM in protecting ALT telomeres and ALT cell viability. However, when we inactivated BLM in FANCM knockout (KO) telomerase positive and non-ALT HCT116 cells, cell viability was also drastically decreased (Fig 7A). These data suggest that the synthetic lethality interaction of BLM and FANCM is not only due to their cooperative functions at ALT telomeres and is likely related to a more general role in coping with replication stress. Our findings that BLM and FANCM play distinct roles in maintaining stability of AT-rich sequences at CFSs may also contribute to the synthetic lethality interactions of these two proteins.

We tested further whether the helicase activity of BLM is important for cell viability of FANCM deficient cells. We expressed BLM-WT or BLM-KD helicase mutant in FANCM WT or FANCM KO HCT116 cells, followed by inactivation of endogenous BLM by shRNAs. Loss of BLM helicase activity drastically reduces cell viability of FANCM KO HCT116 cells while having minimal effect on FANCM WT cells (Fig 7B), suggesting that the BLM helicase activity is essential for cells to survive when FANCM is deficient in non-ALT cells.

## Discussion

CFSs are hotspots for chromosomal rearrangement and are often mapped to breakpoints in cancer cells [2, 44]. We identified a new role of BLM in protection of CFSs and showed that the helicase activity of BLM and ATR-mediated phosphorylation of BLM are important for maintaining stability of AT-rich sequences at CFSs. We also showed that the role of BLM in preventing DSB formation and mitotic recombination at CFS-derived AT-rich sequences is non-overlapping with that of FANCM. The synthetic lethality interaction of BLM and FANCM is observed in both ALT and non-ALT cells and loss of BLM helicase activity in FANCM deficient cells leads to cell death. These studies reveal distinct and cooperative functions of BLM helicase and FANCM translocase to maintain genome stability at CFSs. The role of BLM in the protection of CFSs would likely contribute to its tumor suppression function.

### BLM protects stability of AT-rich sequences derived from CFSs

CFS instability is caused by multiple mechanisms and the presence of AT-rich sequences which are prone to forming secondary structures is one of the causes for CFS expression [45]. Upon replication stress, forks are often stalled at AT-rich sequences at CFSs, leading to fork collapse and DSB formation [18, 19, 21]. We showed that BLM is important for preventing DSB formation and mitotic recombination at AT-rich sequences derived from CFSs.

In the HR-Flex reporter, if DSBs are generated at Flex1 and are repaired by HR, EGFP open reading frame would be restored to produce green cells [21]. Our results showed that inactivation of BLM leads to increased HR-mediated mitotic recombination at Flex1. BLM functions together with DNA2 in end resection during HR [33, 34] and impaired function of BLM is expected to reduce HR efficiency. However, besides BLM/DNA2, Exo1 is also involved in long-range end resection and thus BLM is not absolutely required for HR [33]. Thus, elevated mitotic recombination detected in our reporter in BLM deficient cells indicates that the effect caused by increased DSB formation at Flex1 exceeds the effect of HR reduction due to end resection defect caused by loss of BLM. The role of BLM in protecting CFS-AT is likely underestimated by using the HR-Flex reporter. γH2AX ChIP analysis indicated that DSB formation is increased at Flex1 when BLM is depleted. Therefore, these studies suggest that BLM plays an important role in protecting AT-rich sequences derived from CFSs and suppressing DSB formation at these structure-forming DNA sequences.

BLM is a 3’ to 5’ helicase [27]. Biochemically, BLM is incapable to unwind duplex DNA from blunt-ended terminus or from an internal nick [29]. However, BLM unwinds the bubble and X-structure substrates much more efficiently than 3’-tailed duplex. BLM also efficiently unwinds G4 DNA structures. BLM is thus considered as a DNA structure-specific helicase. CFS-derived AT-rich sequences are also predicted to form strong secondary structures by the Mfold program [S4 Fig, [18, 46]]. We propose that BLM plays an important role in unwinding DNA secondary structures at CFS-derived AT-rich sequences. Upon replication stress, DNA secondary structures would form at CFS-derived AT-rich sequences and stall DNA replication, leading to fork collapse and DSB formation (Fig 7C). Removing DNA secondary structures by BLM unwinding activity would thus prevent fork stalling and DSB formation. The requirement of BLM helicase activity for preventing DSB formation and mitotic recombination at Flex1 supports this model.

### BLM and FANCM play distinct roles and function together to protect CFS-derived AT-rich sequences

We showed that FANCM is important for maintaining stability of CFS-AT sequences in a manner dependent on its translocase activity [23]. FANCM interacts with BLM through RMI1 and this interaction is important for repairing interstrand crosslinks (ICLs) [41]. We showed that the FANCM-MM2 mutant defective for BLM interaction inhibits Flex1-induced mitotic recombination to a similar level as FANCM-WT, suggesting that the role of BLM in suppression of Flex1 instability is not due to its interaction with FANCM. We further showed that mitotic recombination and DSB formation at Flex1 are both increased when BLM and FANCM are simultaneously inactivated compared to BLM or FANCM single deficient cells, suggesting that the role of BLM in preventing instability at Flex1 is distinct and non-overlapping with that of FANCM. These studies support the model that BLM uses mechanisms different from FANCM to protect CFS-AT sequences.

FANCM contains ATP-dependent branch point translocase activity and promotes replication fork regression [47, 48]. We propose that FANCM uses its fork remodeling activity to promote fork regression to remove DNA secondary structures at Flex1 during replication and upon replication stress [23], whereas BLM may directly bind to loops or other secondary structures formed at CFS-AT vicinity to unwind these structures (Fig 7C). Along this line, it has been shown by single molecule FRET analysis that BLM binds to single-stranded DNA at the side of G4 structures to unfold G4 [49]. Unwinding activity mediated by BLM and fork regression activity promoted by FANCM may complement to each other to effectively remove DNA secondary structures formed at AT-rich sequences. On the other hand, BLM can also catalyze replication fork regression [50]. An alternative mechanism could be that FANCM initiates fork reversal to remove DNA secondary structures at forks, but more extensive fork regression may need BLM helicase activity coupled with decatenation activity of Topo IIIα that is bound with BLM [51, 52].

Loss of FANCM or BLM activity both increases mitotic recombination at Flex1, suggesting that HR is used to repair DSBs accumulated at CFS-derived AT-rich sequences when either FANCM or BLM branch is deficient. In support of this, we showed that CtIP and BRCA1 are required for mediating increased mitotic recombination not only when FANCM is defective [23], but also when BLM function is impaired (S5 Fig). Therefore, HR backs up both FANCM and BLM pathways for protecting structure-prone AT-rich sequences. However, when both FANCM and BLM are defective, substantial DSBs are accumulated and HR may not be sufficient to repair all DSBs, thereby leading to cell death.

### Synthetic lethality interaction of FANCM and BLM

It has been described that FANCM and BLM are synthetic lethal in ALT cells, which is caused by impaired telomere replication due to FANCM deficiency, combined with impaired HR function when BLM is defective [43]. We showed that simultaneous inactivation of BLM and FANCM in non-ALT cells also causes cell death, suggesting that the synthetic lethality interaction of FANCM and BLM is not limited to ALT cells and the concerted activities of FANCM and BLM critical for maintaining cell viability maybe more than their involvement in the protection of ALT telomeres.

Our study demonstrates that both FANCM and BLM are important for maintaining stability of structure-forming AT-rich sequences derived from CFSs. We propose that the non-overlapping functions of BLM and FANCM to resolve DNA secondary sequences at replication forks could be one of the important causes for their synthetic lethality interactions (Fig 7C). DNA secondary structures can arise not only at AT-rich sequences in CFSs but also at other structure-prone DNA sequences such as G4s. Computational study predicts more than 700,000 G4 structures existing in the human genome [53]. If the cooperative functions of BLM and FANCM are required for resolving not only CFS-AT but also other DNA secondary structures that are abundantly present in the human genome, it is not surprising that BLM and FANCM have a synthetic lethality interaction. The role of FANCM and BLM in ALT telomere protection can also be attributed to their functions in maintaining stability of structure-forming DNA sequences present at telomeres. Mammalian telomeres are enriched in guanines and are prone to the formation of G4s [54, 55]. It is proposed that the function of FANCM to prevent replication stalling at telomeres is due to its activity to remove G4 secondary structures during telomere replication [43]. Since BLM unwinds G4 *in vitro* [29, 49], it may also be involved in maintaining G4 stability at telomeres *in vivo*. Meanwhile, we also acknowledge that the role of BLM in HR may additionally contribute to the synthetic lethality interaction of BLM and FANCM. Fork collapse at DNA secondary structures would require HR-mediated repair and replication restart that involve BLM for end resection.

### ATR-mediated phosphorylation of BLM is important for protecting stability of AT-rich sequences at CFSs

We showed that ATR-mediated phosphorylation of BLM is important for maintaining Flex1 stability. It is possible that ATR-dependent phosphorylation of BLM stimulates BLM helicase activity, thereby contributing to Flex1 protection. Alternatively, phosphorylation of BLM by ATR may modulate the way of BLM binding to Flex1, and thus influence Flex1 unwinding by BLM. In this aspect, ChIP analysis showed that the recruitment of the ATR phosphorylation mutant BLM-T99A/T122A to Flex1 is largely unchanged. However, it is still possible that ATR-mediated phosphorylation of BLM may influence the affinity of BLM for direct Flex1 binding after its recruitment or modulate the conformation of BLM in complex with Flex1 resulting in change of unwinding activity.

It was shown that the BLM helicase activity and ATR-mediated phosphorylation of BLM are both required for replication restart upon replication stress, but the underlying mechanisms are not clear [37]. We found that both helicase activity and ATR-mediated phosphorylation are important for maintaining CFS-AT stability. We speculate that the intrinsic activity of BLM to remove DNA secondary structures is also used at other genomic loci such as G4s which are very abundant in the genome. Since maintaining fork stability is important for replication restart, we propose that one mechanism involving BLM helicase activity and ATR-mediated phosphorylation in replication restart is linked to its function in resolving DNA secondary structures at replication forks and preventing fork collapse.

BLM is a multifunctional protein which couples recombinational repair with replication fork protection. Our study reveals its new function in conjugation with FANCM to protect stability of AT-rich sequences in CFSs to maintain CFS stability. This role along with its function in end resection at early steps of HR and dissolution to prevent crossover at late steps of HR [56], may underlie the mechanisms of how BLM contributes to the maintenance of genome stability and prevention of cancer.

## Materials and methods

### Cell culture

Human U2OS, 293T and HCT116 cells were cultured at 37°C in a humidified atmosphere of 5% CO2 in Dulbecco’s modified Eagle’s medium (DMEM) supplemented with 10% fetal bovine serum and 1% penicillin/streptomycin. FANCM knockout HCT116 cells were previously described [57].

### Plasmid construction and shRNA Interference

BLM wild-type and indicated mutants were generated by PCR and subcloned into Babe-puro vectors containing 1x N-terminal Flag-tag or NBLV0051 (Novo Bio) vector containing a 3x N-terminal Flag-tag. FANCM wild-type and FANCM-MM2 mutants were also subcloned into NBLV0051 (Novo Bio) vector containing a 3x N-terminal Flag-tag. Point mutations, small hairpin RNA (shRNA) target site-resistant mutations were generated using the QuikChange Site-directed Mutagenesis Kit (Stratagene). The EGFP-based HR-Flex reporter and HR-Luc reporter containing the 0.34 kb Flex1 and luciferase-derived fragment (Luc), respectively, and pCEP4-Flex1 and pCEP4-Luc plasmids were described previously [21]. EGFP-based HR reporter: HR-16C/AT1 and HR-16C/AT3, containing AT-rich sequences derived from FRA16C reporters were described previously [23].

Silencing of endogenous BLM and FANCM was achieved by retroviral or lentiviral infection using pMKO or pLKO vectors to express corresponding shRNAs [58, 59]. shRNA sequences for BLM (GAGCACAUCUGUAAAUUAAUU) and FANCM (GAACAAGAUUCCUCAUUACUU) were designed by Dharmacon.

### Immunoblotting and antibodies

Western blot analysis was performed as described [60]. Cells were lysed with NETN buffer (20 mM Tris, pH 8.0, 1 mM EDTA, 150 mM NaCl, and 0.5% Nonidet P-40) for 30 min. Cell lysates were boiled in 2xSDS loading buffer and subjected to SDS-PAGE.

Commercial antibodies used include anti-BLM (Bethyl Laboratories, A300), anti-H2AX-S139p (Cell Signaling, #2577), anti-Ras (Santa Cruz Biotechnology, sc-520), anti-FLAG (Sigma, F1804), anti-Ku70 (Santa Cruz Biotechnology, sc-17789), anti-β-actin (Sigma, A5441). Antibodies against FANCM was kindly provided by Dr. Weidong Wang [57].

### Mitotic recombination assay and plasmid stability assay

Spontaneous mitotic recombination assay was described before [21]. Briefly, cells carrying HR-Flex or HR-Luc reporter were pre-sorted by FACS (fluorescence-activated cell sorting) to clear background and cultured for indicated days. The mitotic recombination frequency (EGFP-positive events) then was determined by FACS analysis using a BD Accuri C6 flow cytometer and accompanying data analysis software (CFlow, Becton-Dickinson).

Plasmid stability assay was performed as described [21]. Briefly, Flex1 or Luc containing pCEP4 plasmids carrying Epstein-Barr virus (EBV) replication origins and nuclear antigen (encoded by the EBNA-1 gene) to permit extrachromosomal replication in human cells and a hygromycin marker [21], were transfected into the cells expressing wide-type or BLM mutants by lipofectamine 2000 (Thermo Fisher Scientific), followed by hygromycin selection. The endogenous BLM was knocked down by shRNA, and cells were cultured in the absence of hygromycin for 10 days. Then hygromycin was re-introduced into the medium to determine the percentage of cells retaining the plasmids. Sequencing of Flex1 on pCEP4-Flex1 was performed after propagating pCEP4-Flex1 10 days in U2OS cells with or without expressing BLM-shRNAs.

### Growth curve and cell viability assay

Cell proliferation was determined by counting cells every 24 hours using hemocytometer and normalized to the first day. Cell viability was shown by colony formation. HCT116 and its derivative cell lines were plated at 5000 cells per 10 cm plate and were grown in complete media for two weeks. Cell colonies were fixed with cold methanol and stained with 1% crystal violet.

### Chromatin immunoprecipitation (ChIP) assay

ChIP assays were performed as described previously [21]. Briefly, cells were subjected to either no treatment or treatments with APH, followed by cross-linking with 1% formaldehyde for 10 min at room temperature. The cross-linking reaction was terminated by adding glycine to a final concentration of 125 mM and incubating for 5 min. After washing twice with cold PBS, cells were resuspended in lysis buffer (1% SDS, 10 mM EDTA, 50 mM Tris.HCl, pH 8.1) supplemented with protease inhibitor cocktail (“PIC”, cOmplete, Roche) and the samples were subject to sonication to break chromatin into fragments with an average length of 0.2–1.0 kb. The supernatants were collected by centrifugation and were pre-cleared with Protein A/G Sepharose beads (Amersham Biosciences). Immunoprecipitation (IP) was performed using H2AX-S139p antibody (Cell Signaling #2577) or Anti-Flag antibody (Sigma F1804) followed by washing with TSE I (0.1% SDS, 1% Triton X-100, 2 mM EDTA, 20 mM Tris.HCl, pH 8.1, 150 mM NaCl), TSE II (0.1% SDS, 1% Triton X-100, 2 mM EDTA, 20 mM Tris.HCl, pH 8.1, 500 mM NaCl), buffer III (0.25 M LiCl, 1% NP-40, 1% deoxycholate, 1 mM EDTA, 10 mM Tris.HCl, pH 8.1), and TE. The protein-DNA complex was then eluted from beads by elution buffer (1% SDS, 0.1 M NaHCO_3_), and cross-linking was reversed by adding in 4 μl of 5 M NaCl and incubating at 65°C for 6 hr, followed by proteinase K digestion for 2 hr at 42°C. DNA was extracted by QIAquick kit (QIAGEN) according to the manufacture instructions.

For ChIP at Flex1 in the HR reporter stably integrated in the genome or at AT-rich sequences in the endogenous FRA3B locus, recovered DNA was analyzed by quantitative PCR (qPCR) and the readout was normalized to the vector control which is set as 1. GAPDH locus was used as a control to show the specificity of protein binding to Flex1. The primers used for ChIP at Flex1 in HR reporter: P1F 5’CTCCAATTCGCCCTATAGTGAGTCGTATTA, P1R 5’TTACTTGTACAGCTCGTCCATGC, P2F 5’GGCAGTACATCAATGGGCGTG, and P2R 5'CCTTTAGTGAGGGTTAATTGCGCG; at AT-rich sequences in FRA3B: 3B-P1F 5’TTAGCCTACTTCAGGGTTTCT and 3B-P1R 5’TGGAGAGGTTACTACTGGCA; and at GAPDH: GAPDH-F 5’CCCTCTGGTGGTGGCCCCTT and GAPDH-R 5’GGCGCCCAGACA CCCAATCC.

For γH2AX ChIP at Flex1 or Luc surrounding regions on the pCEP4-Flex1 or pCEP4-Luc plasmids, recovered DNA was amplified by regular PCR or qPCR with primers 5’TCAGGGGGAGGTGTGGGAGG and 5’GCAGTCCACAGACTGCAAAG. Regular PCR was programmed as preheat at 95°C for 3 min followed by 35 cycles of 95°C 15 sec, 50°C 15 sec, 70°C 30sec. Regular PCR products were resolved by 1.5% agarose DNA gel [21]. Alternatively, qPCR was performed for γH2AX ChIP.

### Fragile site analysis

Fragile site analysis using fluorescence in situ hybridization (FISH) was performed as described [21]. Briefly, cells were treated with 0.4 μM APH for 18 hr to induce CFS expression, followed by incubation of 0.1 μg/ml colcemid at 37°C for 45 min as described in the standard method for chromosome preparations. Collected cells were resuspended in 75 mM KCl hypotonic solution pre-warmed to 37°C and incubated at 37°C for 30 min, followed by several changes of fixative solution (3:1 methanol/acetic acid). Cells were dropped onto slides and incubated for 2 hr at 60°C prior to FISH analysis. FISH experiments were performed according to standard protocols [61]. Green 5-Fluorescein dUTP-labeled probes 264L1 (FRA16D) from RPCI-11 human BAC library (Empire Genomics) were used as probes for FISH analyses. Chromosomes were counterstained with DAPI.

### Quantification and statistical analysis

All the statistical data are the results of at least three independent experiments and presented as mean±SD. Numerical data for all figures are included in S1 Data.

## Supporting information

S1 Fig DSBs are formed at Flex1 when BLM is deficient. ChIP analysis of γH2AX at Flex1 in pCEP4-Flex1 or Luc in pCEP4-Luc was performed by qPCR using samples described in Fig 2C (A); Fig 3C (B).; Fig 4C (C); and Fig 6C (D). Three experiments were performed with error bars representing standard deviation (SD). **refers to P value < 0.01 and n.s. is not significant.
(TIF)

S2 Fig DSBs are formed at endogenous FRA3B locus when BLM is depleted. Anti-γH2AX ChIP analysis at endogenous FRA3B locus was performed by qPCR in U2OS cells expressing BLM shRNA or vector (Ctrl) upon APH treatment (0.4 uM, 24 hr) using indicated primers (left). ChIP value in Ctrl is set as 1 for normalization. BLM expression is indicated by Western analysis (right).
(TIF)

S3 Fig The FANCM-MM2 mutant is defective in BLM binding. Flag-FANCM-WT or Flag-FANCM-MM2 was expressed in 293T cells, and the interaction of Flag-FANCM and Flag-FANCM-MM2 with endogenous BLM was examined by IP of Flag followed by anti-BLM Western.
(TIF)

S4 Fig AT-rich sequences derived from CFSs can form DNA secondary structures. Prediction of DNA secondary structures of Flex1, 16C/AT1 and 16C/AT3 with highest stability was performed by the Mfold Program (http://unafold.rna.albany.edu/?q=mfold, Nucleic Acids Res. 2003; 31(13): 3406–3415).
(TIF)

S5 Fig CtIP and BRCA1 are required for increased mitotic recombination when BLM is depleted. U2OS (HR-Flex) cells expressing shRNAs for CtIP or BRCA1, or control vector (Ctrl), were infected with lentiviruses encoding shRNAs for BLM. Spontaneous mitotic recombination was examined 8 days after infection with a control without expressing shRNAs. Western blots are shown for indicated proteins.
(TIF)

S1 Data Numerical data for Figures. (XLSX)
