# Supplementary figures and images for: BLM prevents instability of structure-forming DNA sequences at common fragile sites
Source: PLoS Genet. 2018 Nov 29;14(11):e1007816. doi: 10.1371/journal.pgen.1007816 (PMC6289451; doi:10.1371/journal.pgen.1007816)

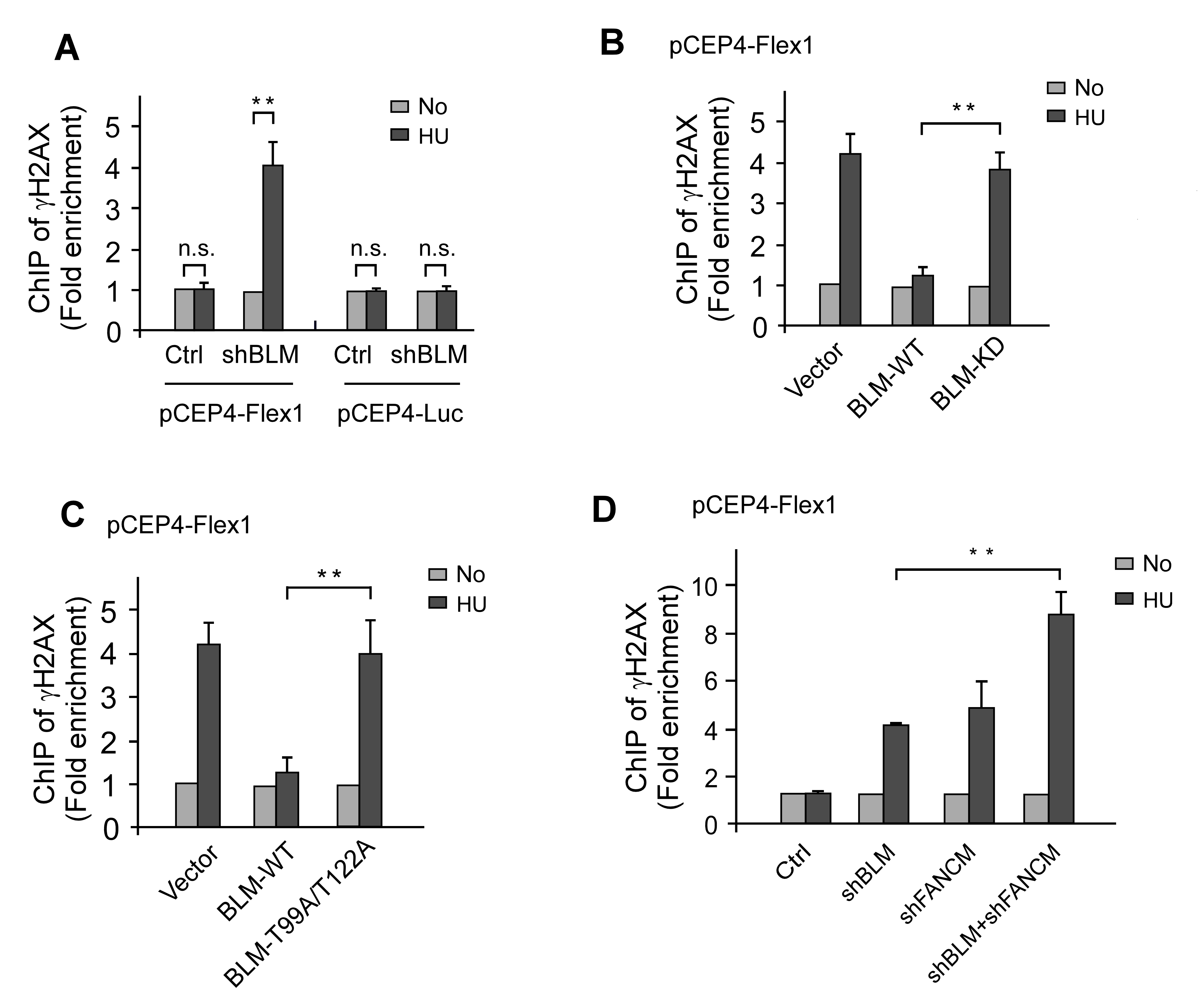

Supplement: S1 Fig — ChIP analysis of γH2AX at Flex1 in pCEP4-Flex1 or Luc in pCEP4-Luc was performed by qPCR using samples described in Fig 2C (A); Fig 3C (B).; Fig 4C (C); and Fig 6C (D). Three experiments were performed with error bars representing standard deviation (SD). **refers to P value < 0.01 and n.s. is not significant. (TIF) [file pgen.1007816.s001.tif]

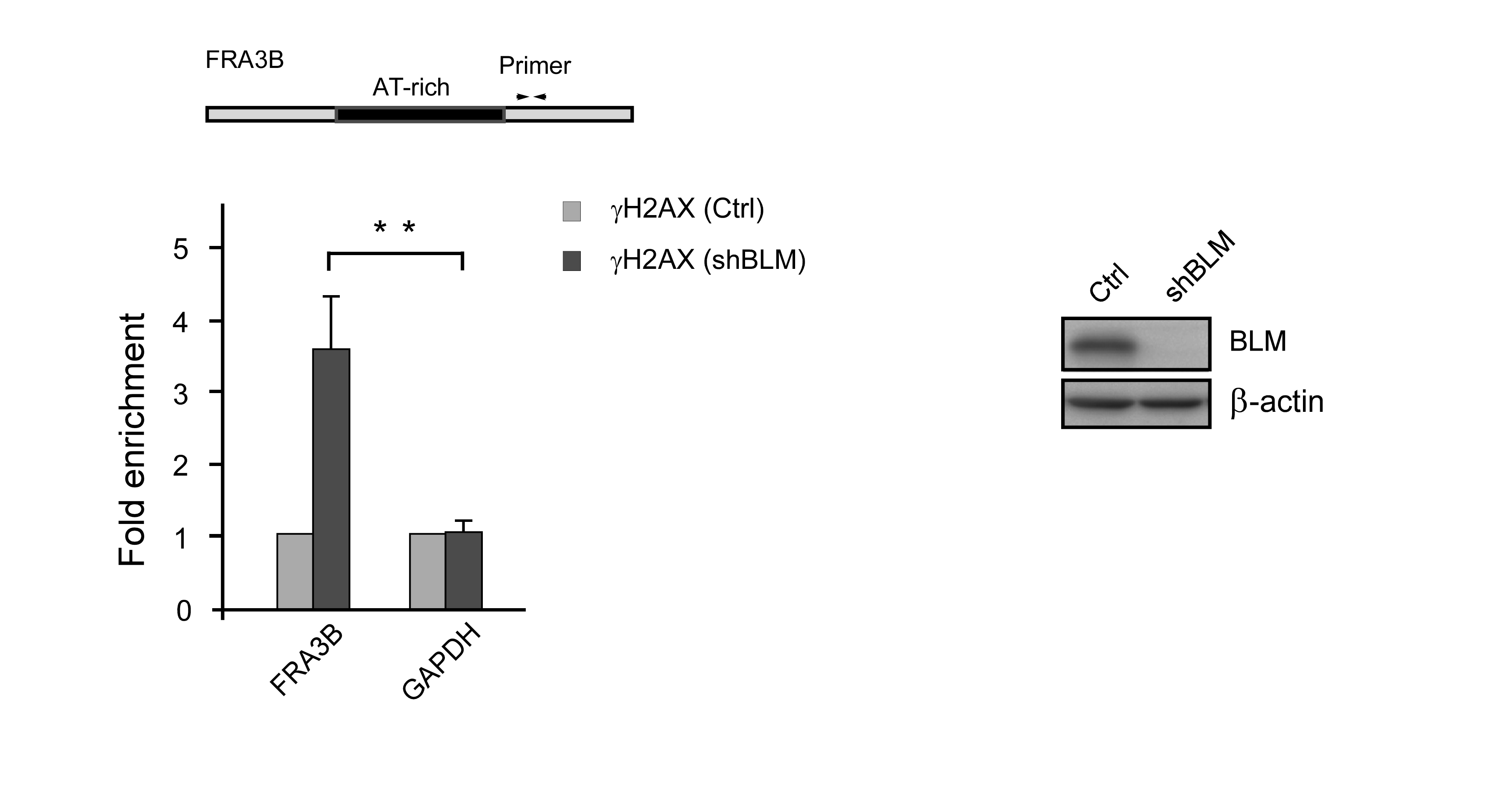

Supplement: S2 Fig — Anti-γH2AX ChIP analysis at endogenous FRA3B locus was performed by qPCR in U2OS cells expressing BLM shRNA or vector (Ctrl) upon APH treatment (0.4 uM, 24 hr) using indicated primers (left). ChIP value in Ctrl is set as 1 for normalization. BLM expression is indicated by Western analysis (right). (TIF) [file pgen.1007816.s002.tif]

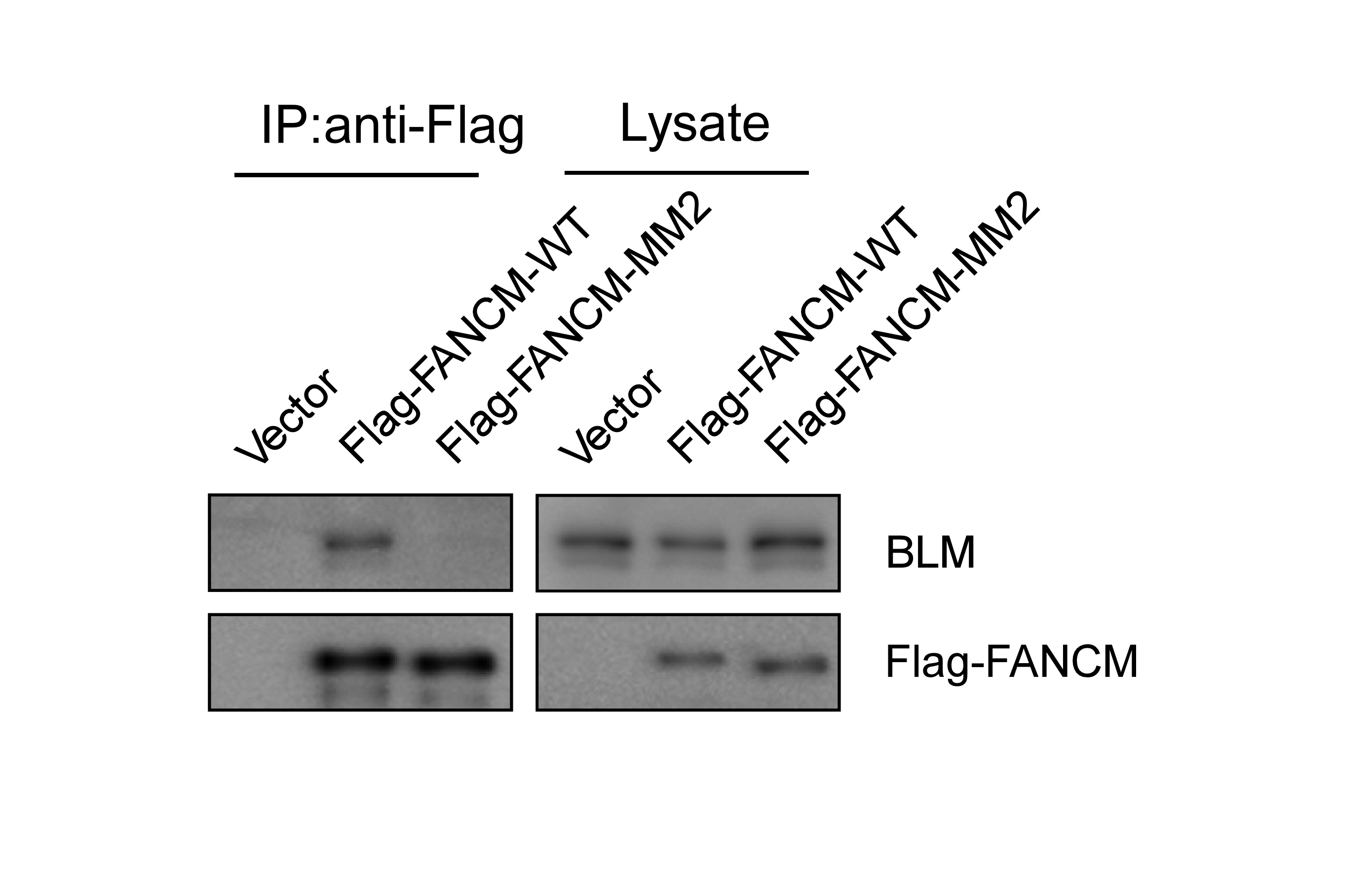

Supplement: S3 Fig — Flag-FANCM-WT or Flag-FANCM-MM2 was expressed in 293T cells, and the interaction of Flag-FANCM and Flag-FANCM-MM2 with endogenous BLM was examined by IP of Flag followed by anti-BLM Western. (TIF) [file pgen.1007816.s003.tif]

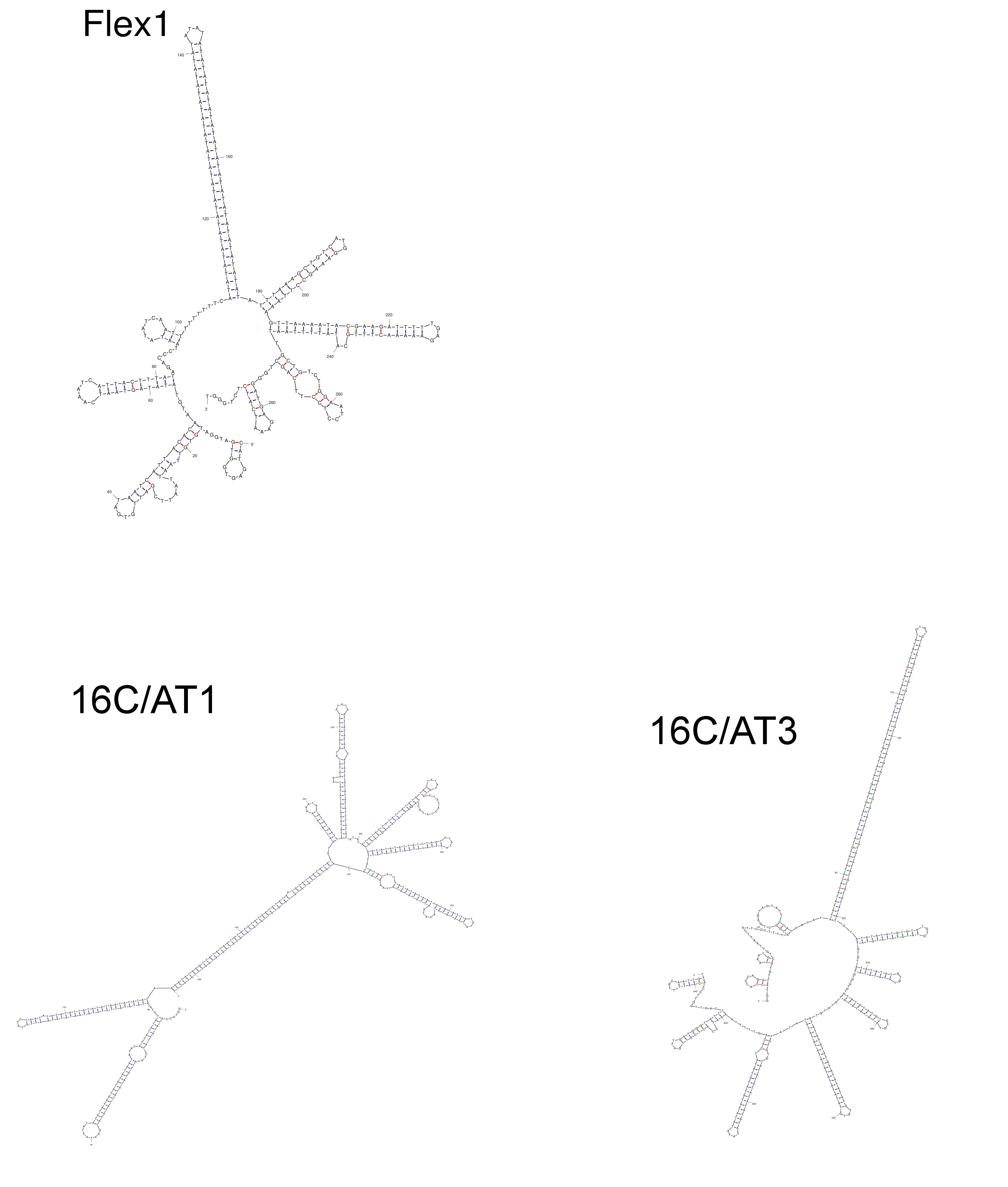

Supplement: S4 Fig — Prediction of DNA secondary structures of Flex1, 16C/AT1 and 16C/AT3 with highest stability was performed by the Mfold Program (http://unafold.rna.albany.edu/?q=mfold, Nucleic Acids Res. 2003; 31(13): 3406–3415). (TIF) [file pgen.1007816.s004.tif]

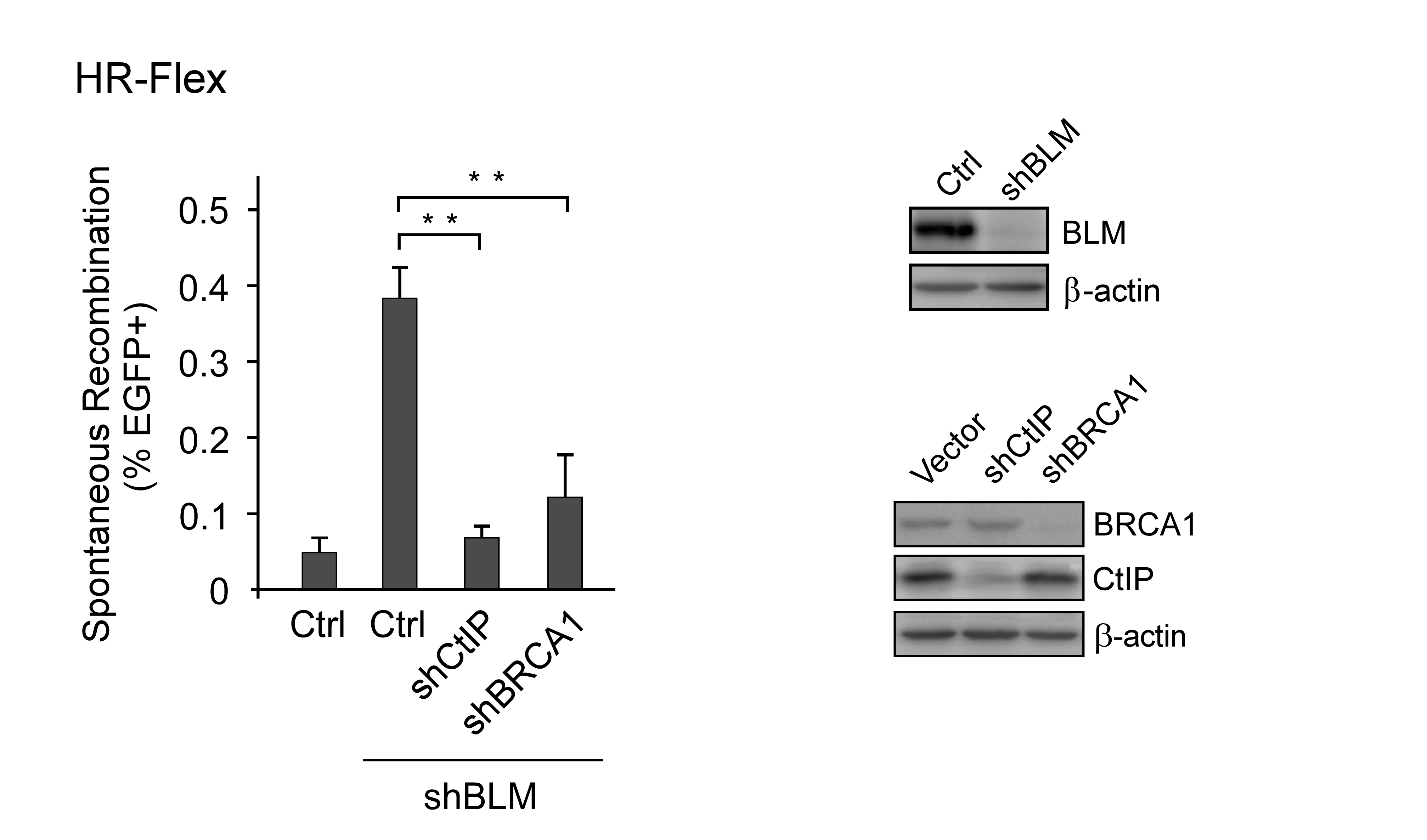

Supplement: S5 Fig — U2OS (HR-Flex) cells expressing shRNAs for CtIP or BRCA1, or control vector (Ctrl), were infected with lentiviruses encoding shRNAs for BLM. Spontaneous mitotic recombination was examined 8 days after infection with a control without expressing shRNAs. Western blots are shown for indicated proteins. (TIF) [file pgen.1007816.s005.tif]
